# Insights from a Multi-Omics Integration (MOI) Study in Oil Palm (*Elaeis guineensis* Jacq.) Response to Abiotic Stresses: Part Two—Drought

**DOI:** 10.3390/plants11202786

**Published:** 2022-10-20

**Authors:** André Pereira Leão, Cleiton Barroso Bittencourt, Thalliton Luiz Carvalho da Silva, Jorge Cândido Rodrigues Neto, Ítalo de Oliveira Braga, Letícia Rios Vieira, José Antônio de Aquino Ribeiro, Patrícia Verardi Abdelnur, Carlos Antônio Ferreira de Sousa, Manoel Teixeira Souza Júnior

**Affiliations:** 1 Embrapa Agroenergia, Brasília 70770-901, DF, Brazil; 2 Graduate Program of Plant Biotechnology, Federal University of Lavras, Lavras 37200-000, MG, Brazil; 3 Embrapa Meio Norte, Teresina 64006-245, PI, Brazil

**Keywords:** transcriptomics, proteomics, metabolomics, integratomics, abiotic stress, African oil palm

## Abstract

Drought and salinity are two of the most severe abiotic stresses affecting agriculture worldwide and bear some similarities regarding the responses of plants to them. The first is also known as osmotic stress and shows similarities mainly with the osmotic effect, the first phase of salinity stress. Multi-Omics Integration (MOI) offers a new opportunity for the non-trivial challenge of unraveling the mechanisms behind multigenic traits, such as drought and salinity resistance. The current study carried out a comprehensive, large-scale, single-omics analysis (SOA) and MOI studies on the leaves of young oil palm plants submitted to water deprivation. After performing SOA, 1955 DE enzymes from transcriptomics analysis, 131 DE enzymes from proteomics analysis, and 269 DE metabolites underwent MOI analysis, revealing several pathways affected by this stress, with at least one DE molecule in all three omics platforms used. Moreover, the similarities and dissimilarities in the molecular response of those plants to those two abiotic stresses underwent mapping. Cysteine and methionine metabolism (map00270) was the most affected pathway in all scenarios evaluated. The correlation analysis revealed that 91.55% of those enzymes expressed under both stresses had similar qualitative profiles, corroborating the already known fact that plant responses to drought and salinity show several similarities. At last, the results shed light on some candidate genes for engineering crop species resilient to both abiotic stresses.

## 1. Introduction

The palm oil industry established itself in places around the world where the oil palm (*Elaeis guineensis* Jacq.), the number one source of vegetable oil consumed today, exists in its natural environment of high rainfall throughout the year. Those places are in an area of 5° latitude from the Equator line, and as one moves further north or south in the tropical belt, one experiences long periods of drought throughout the year when oil palm does not receive the physiological water level required to maintain productivity. Consequently, the oil palm grower must embrace artificial irrigation, together with the production cost linked to it, not to mention the risk of facing problems of soil salinization.

A recent report by the UN Convention to Combat Desertification, entitled Drought in Numbers 2022—Restoration to Readiness and Resilience, brings to light the dimension of the damages caused by this abiotic stress on the planet and shows to some extent what is needed to overcome this problem and sustainably ensure food security [1]. Drought stress is one of the most severe environmental stresses affecting agriculture worldwide. The efficient and effective use of genetics and biotechnology is among the most proactive interventions necessary to mitigate the effects of water deprivation on plant productivity [2].

Plant response to drought is a complex biological system that needs investigation via an integrative approach that must involve both drought-tolerant and intolerant genotypes. Resistance to drought in plants results from the dynamic interaction of various parts of this system that need deep understanding in order to enable the design of approaches for the effective vertical or horizontal transfer of such traits to drought intolerant genotypes [2,3]. A Multi-Omics Integration (MOI) study [4,5,6], made viable due to the recent massive generation of genomics, transcriptomics, proteomics, and metabolomics datasets, is helping the non-trivial challenge of unraveling the mechanisms behind this multigenic trait, and mapping not only resistance genes but also their hot spots for precision genome editing [7].

Plant responses to drought and salinity stresses show several similarities, especially in the first phase of the latter, the osmotic effect [8,9,10]. Distinct studies showed the existence of genes able to promote resistance to more than one abiotic stress at once. For instance, Brini et al. [11] showed that the transgenic *Arabidopsis thaliana* plants overexpressing two distinct genes from wheat were much more resistant to both stresses than the isogenic wild-type plants. The same was true for Feng et al. [12], who overexpressed a gene from pepper in *A. thaliana* and showed that this gene positively regulates heat, salt, and drought resistance in pepper. In another example, Zhang et al. [13] overexpressed three genes from sweet potato in sweet potato and obtained genetically modified plants tolerant to salt and drought stresses.

Jha et al. [14], Zargar et al. [15], Muthuramalingam et al. [16], and Bittencourt et al. [17] are a few examples of recent reports dealing with the use of MOI approaches toward understanding plant responses to these abiotic stresses. The current study is a step forward in our research activities on characterizing the biochemical and molecular response of oil palm to abiotic stress. After carrying out a comprehensive, large-scale, single-omics analysis (SOA) and an MOI analysis of the metabolome, transcriptome, and proteome profiles on the leaves of young oil palm plants submitted to salinity stress [17], we here report a similar study characterizing the response of this oilseed crop to water deprivation. Furthermore, the differentially expressed (DE) molecules responsive to both drought (from this study) and salt (from [17]) underwent additional MOI analysis and correlation study to identify the commonalities and differences in the response of such plants to both stresses.

## 2. Results

### 2.1. Oil Palm Transcriptome under Drought Stress

When comparing drought-stressed plants against the control ones, the pairwise differential expression analysis revealed that out of the 43,551 proteins from the oil palm genome [18,19], 8421 (19.34%) were differentially expressed (DE) at False Discovery Rate (FDR) ≤ 0.05; being 4262 upregulated (Log_2_(FC) > 0) and 4159 downregulated (Log_2_(FC) < 0) (Supplementary Table S1).

This group of 8421 DE protein sequences was submitted to analysis in the BlastKOALA annotation tool for the K number assignment, revealing 4133 DE proteins with K number and 1955 with Enzyme Commission (EC) numbers. This set of 1955 DE enzymes underwent gene ontology analyses to classify them accordingly to biological process (BP), molecular function (MF), and cellular component (CC).

**Figure 1:**
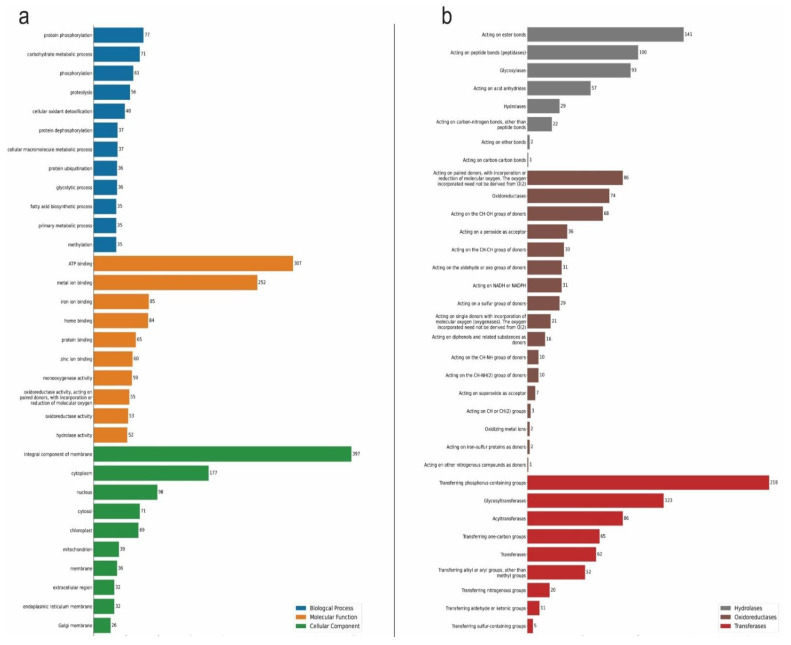
Gene Ontology (GO) annotation classification statistics graph from full-length transcriptome in the leaves of young oil palm plants under drought stress. Classified accordingly to biological process, cellular component, and molecular function (**a**); and to chemical reactions by which proteins are classified according to EC (**b**). Only the most populated groups per GO term, and only the three prevalent classes of enzymes are shown. Numbers represent the amount of positive hits.

The BP subgroups with more representatives were protein phosphorylation, followed by carbohydrate metabolic process, phosphorylation, proteolysis, and cellular oxidant detoxification. For MF, the most populated subgroups were ATP binding, metal ion binding, iron ion binding, and heme binding. Finally, for the CC subgroup, the integral component of the membrane came in first, followed by the cytoplasm, nucleus, and cytosol components (Figure 1a).

Furthermore, the DE enzymes underwent classification according to the EC number, a numerical classification scheme for enzymes based on the chemical reaction. The first classification level involves a general type of enzyme-catalyzed reaction that ranges from one to six, and the oxidoreductases (EC 1), transferases (EC 2), and hydrolases (EC 3) classes dominated. Enzymes acting on paired donors, with the incorporation or reduction in molecular oxygen (EC 1.14) was the most populated group among the subclasses of oxidoreductases, with 86 hits. The most representative subclass of transferases included those with a function of transferring phosphorus-containing groups, glycosyltransferases, and acyltransferases, with 218, 123, and 86 hits, respectively. Finally, the hydrolases had the following subclasses standing out with over 50 hits: compounds involved and acting on ester bonds, acting on peptide bonds (peptidases), glycosylases, and acting on acid anhydrides (Figure 1b).

### 2.2. Oil Palm Proteome under Drought Stress

It was possible to identify with high confidence (FDR ≤ 0.01) a total of 3659 and 3824 peptides in control and stressed samples, respectively, which infers up to 1859 protein entries from *E. guineensis* proteome (Uniprot) in both conditions (Table 1).

Seven hundred and seventy-nine proteins were inferred from more than four peptides and five hundred and ten had at least one proteotypic peptide observation. All peptides and proteins identified and the list of 1085 proteins according to the maximum parsimony criterion are presented in Supplementary Tables S2–S4. The control and salt-stressed treatments shared 706 protein identifications, while 76 and 152 proteins were uniquely detected in control and stressed samples, respectively (Supplementary Tables S5 and S6). Fifty-six proteins showed statistically significant differences in their abundance between stressed and control samples; among them, there were thirty-five proteins upregulated and twenty-one downregulated (Supplementary Table S7). Our differential abundance analysis considered proteins identified at least in two replicates in each condition.

A group of 284 proteins that attended to the statistical criteria of PatternLab V software underwent functional annotation and MOI analyses, being 76 found exclusively in the control plants, 152 found only in the drought-stressed plants, and 56 showed statistically significant differences in their abundance between the two treatments used. Those 284 DE proteins underwent analysis in the BlastKOALA annotation tool, revealing 215 DE proteins with K numbers and 131 with EC numbers.

**Figure 2:**
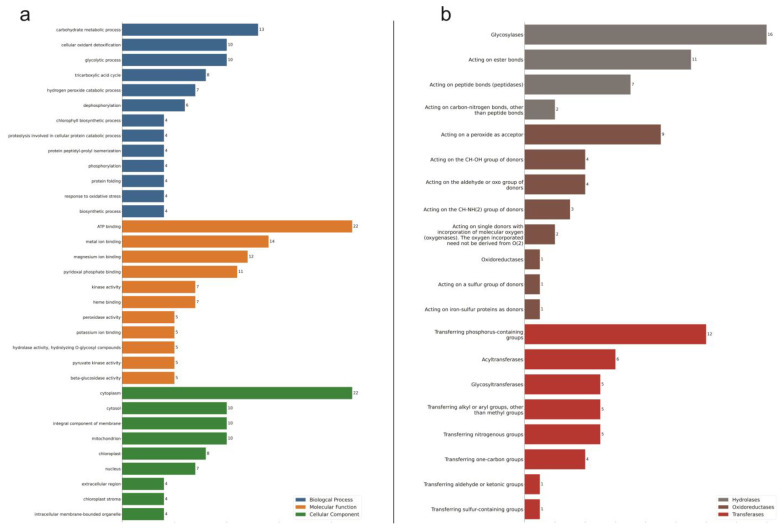
Gene Ontology (GO) annotation classification statistics graph from proteome in the leaves of young oil palm plants under drought stress. Classified accordingly to biological process, cellular component, and molecular function (**a**); and to chemical reactions by which proteins are classified according to EC. Only the most populated groups per GO term, and only the three prevalent classes of enzymes are shown (**b**). Numbers represent the amount of positive hits.

This set of 131 DE enzymes underwent gene ontology analyses to classify them accordingly to biological process (BP), molecular function (MF), and cellular component (CC). The BP top subgroups were carbohydrate metabolic processes, followed by cellular oxidant detoxification and glycolytic process, with 13, 10, and 10 hits, respectively. For MF, the proteins were mainly from subgroups of ATP binding, metal ion binding, magnesium ion binding, and pyridoxal phosphate binding, all with over ten hits. Finally, the CC of the cytoplasm came in first, followed by the cytosol, integral component of membrane, and mitochondrion, all with over ten hits (Figure 2a).

The prevalent chemical reactions according to EC were Oxireductases (EC 1), transferases (EC 2), and hydrolases (EC 3) classes. In the subclasses of oxidoreductases, the top group was acting on a peroxide as an acceptor, followed by acting on the CH-OH group of donors and acting on the aldehyde or oxo group of donors. The most representative subclass of transferases class was the one with transferring phosphorus-containing groups (Figure 2b).

The results from transcriptome and proteome single-omics analysis (SOA), when comparing drought-stressed young oil palm plants against the control ones, show that the ATP binding came as the number one BP affected by the stress, followed by the metal ion binding process. Meanwhile, transferring phosphorus-containing groups, glycosyltransferases, and acyltransferases were subclasses of prevalent chemical reactions in both SOA (Figure 1 and Figure 2).

### 2.3. Oil Palm Metabolome under Drought Stress

The statistical analysis performed on the MetaboAnalyst has returned 3363 and 2538 peaks in the positive and negative polar fractions, respectively. Ninety-six were up- and seventy-one downregulated in the polar-positive. Two hundred ninety-one were up- and nine downregulated, in the polar-negative.

All 467 differentially expressed peaks (DEPs) were then submitted to functional interpretation via analysis in the MS Peaks to Pathway module, and the combined mummichog and GSEA pathway meta-analysis produced a list of 269 differentially expressed metabolites (DEMs), which underwent analysis in the pathway topology analysis module (Supplementary Table S8). The tyrosine metabolism (map00350); phenylpropanoid biosynthesis (map00940); arginine biosynthesis (map00220); isoquinoline alkaloid biosynthesis (map00950); pyrimidine metabolism (map00240); arginine and proline metabolism (map00330); betalain biosynthesis (map00965); monobactam biosynthesis (map00261); valine, leucine and isoleucine biosynthesis (map00290); pentose phosphate pathway (map00030); aminoacyl-tRNA biosynthesis (map00970); flavonoid biosynthesis (map00941); histidine metabolism (map00340); stilbenoid, diarylheptanoid and gingerol biosynthesis (map00945); riboflavin metabolism (map00740); phenylalanine, tyrosine and tryptophan biosynthesis (map00400); and one carbon pool by folate (map00670) pathways were the most affected pathways with a raw *p* ≤ 0.05; in this order, from top to bottom.

### 2.4. Integrating Oil Palm Transcriptome, Proteome and Metabolome under Drought Stress

**Figure 3:**
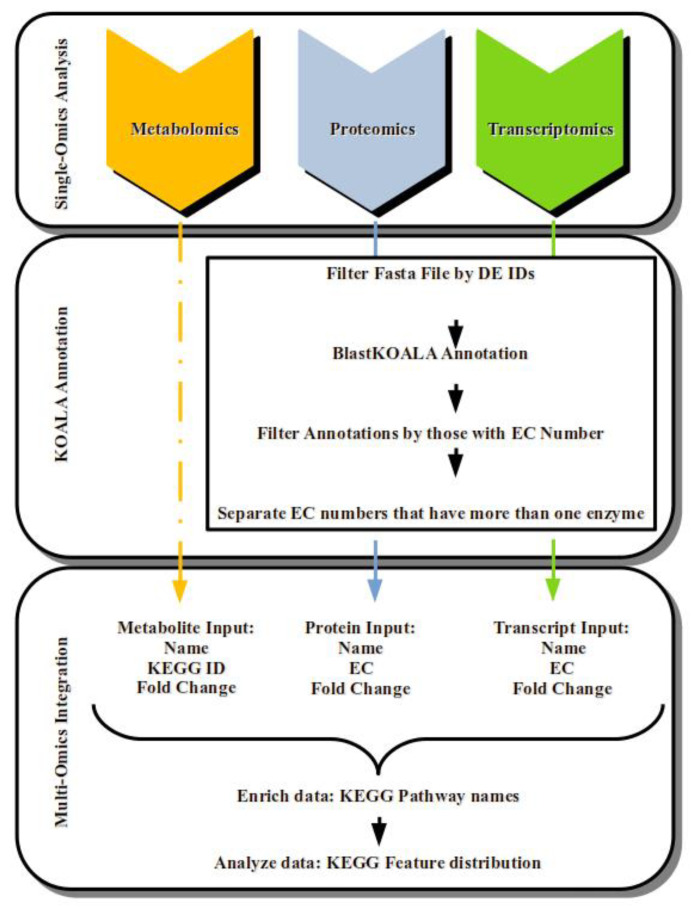
Summarized workflow used to Single-Omics Analysis (SOA), KOALA Annotation, and Multi-Omics Integration (MOI) applied to characterize the transcriptomics, proteomics, and metabolomics datasets generated from the leaves of young oil palm plants submitted to abiotic stress.

The approach used to integrate the datasets from the three omics platforms was a pathway-based mapping approach, similar to that previously used by Bittencourt et al. [17] (Figure 3).

All enzymes (from SOA analysis—transcriptomics or proteomics) and metabolites (from metabolomics SOA analysis), selected as differentially expressed in the leaves of young oil palm plants (stressed/control), underwent MOI analysis.

**Figure 4:**
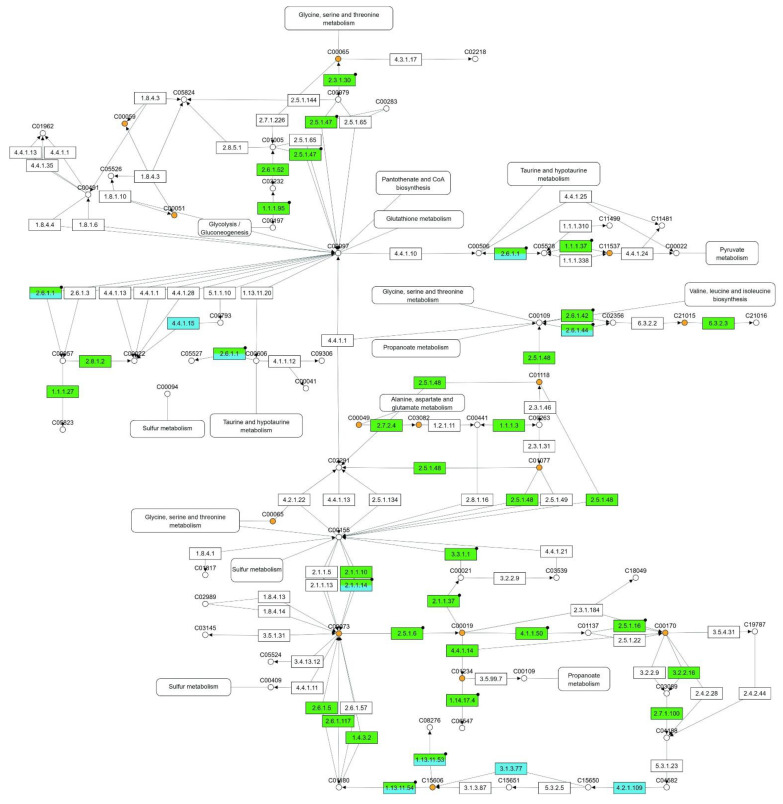
Enzymes (EC number) and metabolites (KEGG Compound number) from the Cysteine and methionine metabolism (map00270) pathway differentially expressed in the apical leaf of young oil palm plants submitted to drought stress. Metabolites differentially expressed are shown as orange circles, metabolites non-differentially expressed are shown as white circles, and enzymes non-differentially expressed are shown as white, and those differentially expressed are shown as green (from transcriptomics), as blue (from proteomics), and green/blue rectangles (from transcriptomics and proteomics).

When applying the Omics Fusion platform to perform the MOI analysis, a group of 56 pathways appeared as affected by drought stress, with at least one molecule differentially expressed in each of the three omics platforms used. Those pathways with ≥20 unique molecules differentially expressed are shown in Table 2. The Cysteine and methionine metabolism (map00270) pathway came first, with 47 molecules from the transcriptome/proteome/metabolome integrative analysis (Figure 4).

### 2.5. Commonalities and Dissimilarity in the Transcriptome, Proteome, and Metabolome Profiles of Young Oil Palm Plants Separately Submitted to Drought and Salinity Stresses

To generate the data regarding the commonalities and dissimilarity in the response of young oil palm plants separately submitted to drought and salinity stresses, all datasets generated in this present study underwent a comparison analysis with the equivalent datasets that had been previously generated and used in Bittencourt et al. [17].

**Figure 5:**
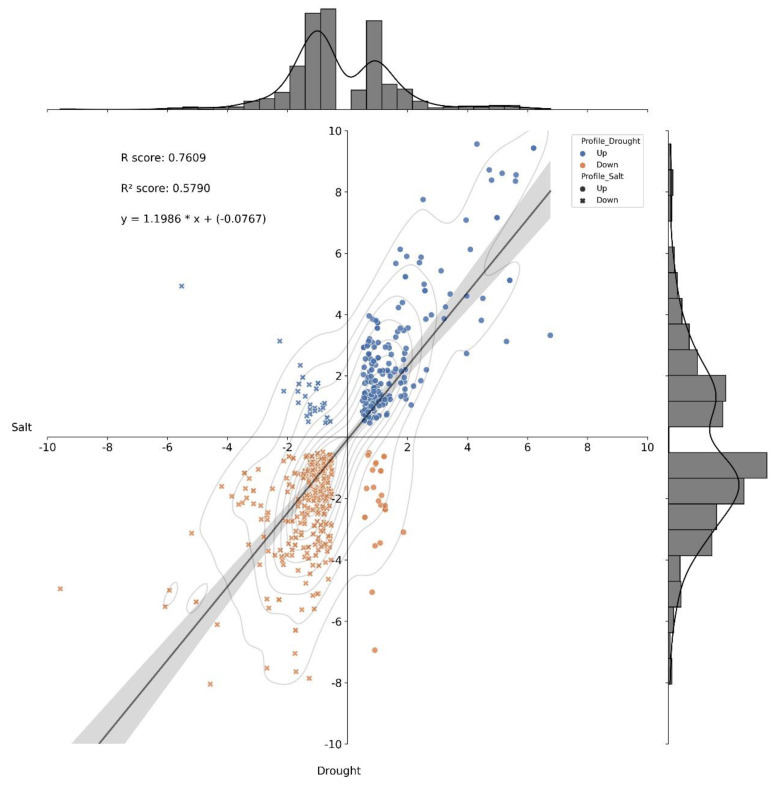
Histogram and correlation analysis of the Log_2_(FC) of differentially expressed enzymes by pairwise comparison of two scenarios—drought and salinity stress. FC—fold change. Dots represent enzymes positively regulated under salt stress, x’s represent enzymes negatively regulated under salt stress. Blue dots and x’s represent enzymes positively regulated under drought-stress, and orange dots and x’s represent enzymes negatively regulated under drought-stress.

**Figure 6:**
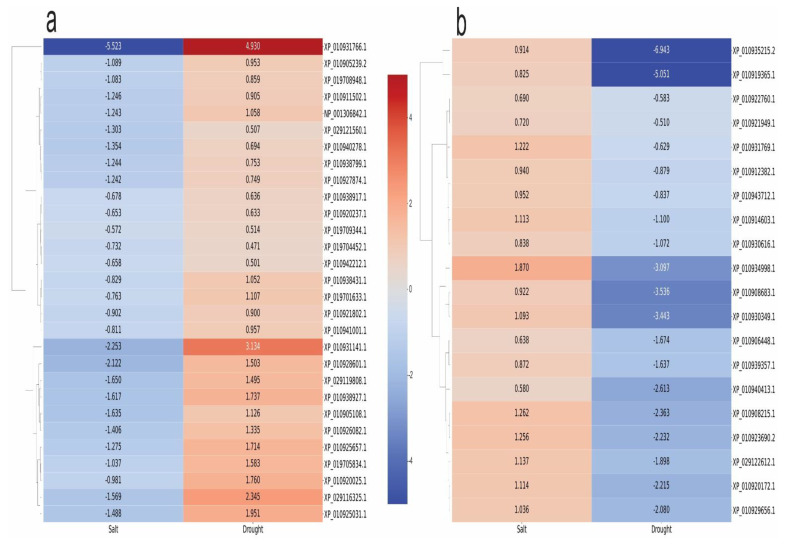
Heatmap of the differentially expressed enzymes from transcriptomics analysis that showed different qualitative profiles in the two scenarios studied—drought and salinity stresses. (**a**)—downregulated in salt and upregulated in drought stress; and (**b**)—upregulated in salt and downregulated in drought stress.

In the transcriptomics analysis, there is a positive correlation between the behavior of the 554 enzymes differentially expressed under drought and salinity stresses (Supplementary Table S9) when 91.15% of the enzymes had the same qualitative profile (upregulated or downregulated), independently of the stress condition (Figure 5). This result is a strong amount of evidence that there are several commonalities regarding gene expression—molecular dynamics—in young oil palm plants submitted separately to these two abiotic stresses. It is also a valuable source of information to further study the osmotic effect. However, 8.85% of those enzymes had different qualitative profiles (Figure 5), where 29 enzymes were downregulated under salt stress and upregulated under drought (Figure 6a), and 20 had the opposite behavior (Figure 6b).

In the case of the proteomics analysis, a group of enzymes were also differentially expressed under both abiotic stresses (Table 3). Most of them had the same qualitative profile independently of the stress condition and a minority had different ones. For instance, enzymes A0A6J0PP21 and A0A6I9S451, two isoforms coded by the same gene (LOC105053549) in the oil palm genome and described as L-ascorbate peroxidases in the KEGG database, were upregulated under drought stress and downregulated almost to no detection under salt stress.

**Figure 7:**
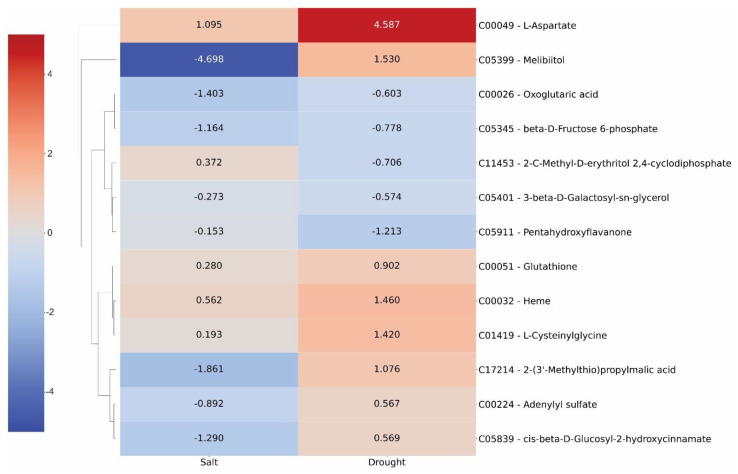
Heatmap of the 13 differentially expressed metabolites that were found in the two scenarios studied—drought and salinity stresses.

In the case of the metabolomics analysis, a group of 13 metabolites was also differentially expressed in both scenarios. Among them, eight had the same qualitative profile, independently of the stress and five had different ones (Figure 7). The metabolite Melibiitol (C05399) was the one that showed the highest quantitative difference in expression, with a Log_2_(FC) of −4.698 (or 3.85% of the initial expression level in the control treatment) under salinity stress and of 1.53 (or 190% on the top of the initial expression level) under drought stress.

At last, when applying the Omics Fusion platform to perform the MOI analysis using only the proteins and metabolites DE in both scenarios, Cysteine and methionine metabolism came first in the list of most affected pathways, with 15 full-length transcripts coding for enzymes, two enzymes (from the proteomics analysis), and two metabolites (Table 4).

## 3. Discussion

Plant response to water deprivation shows several similarities with the first phase of salinity stress, the osmotic effect [8,9,10]. The current study is a step further in a study in our lab that applies different omics platforms to characterize the morphophysiological and molecular responses of young oil palms to two abiotic stresses—salinity and drought [17,20,21,22,23].

The young oil palm plants used in the study by Bittencourt et al. [17] and the ones used in the present study are clones of a specific plant—genotype AM33, a Deli x Ghana from ASD Costa Rica. Moreover, they were of almost similar age, cultivated under the same main environmental variables—as they were submitted to stress almost at the same time in the same greenhouse, and the stresses lasted 12 days in the case of salinity and 14 days in the case of drought, or almost the same time. Consequently, one can say that it is a unique opportunity to use all omics datasets—transcriptomics, proteomics, and metabolomics—from either one of the studies to map the similarities and dissimilarities in the response of those plants to those abiotic stresses. It is important to state that, in the case of salinity stress, plants were under field conditions throughout the entire experiment to ensure that the osmotic effect was due to the excess of salt in the soil and not otherwise [20].

In the present study, single-omics analyses showed that the most affected biological process subcategories in the transcriptome were protein phosphorylation, carbohydrate metabolic, and phosphorylation processes; meanwhile, in the proteome, the most affected ones were carbohydrate metabolism, cellular oxidant detoxification, and glycolytic processes. The transcriptomics and proteomics results, whether under salinity [17] or drought stress (present study), show that the most affected biological process subcategories in the transcriptome and proteome profiles are very much the same in the apical leaf of young oil palm plants.

Stress mitigation results from the action of several distinct proteins, allowing plants to survive under unfavorable conditions, including water deprivation. Post-translation modifications, such as protein phosphorylation, promote changes in those proteins that already existed in the cells before the onset of the stress and play roles important in plant resistance to abiotic stresses [24,25]. Carbohydrate metabolism also plays a role in plant stress perception and signal transduction, besides being a substrate for energy production and a mediator of osmotic regulation and carbohydrate distribution [26].

The number one molecular function affected by drought or salinity stress in the apical leaf of young oil palm plants, revealed by both the transcriptome and proteome profiles obtained here and by Bittencourt et al. [17], was ATP binding. ATP binding proteins (ABPs) have a binding site that allows adenosine-5’-triphosphate (ATP) molecules to interact, and they have roles important in membrane transport and the regulation of various metabolic processes. The ATP-binding cassette (ABC) transporters belong to a large protein family and are present in the membranes of chloroplasts, mitochondria, peroxisomes, and vacuoles. They participate in several biological processes of regulation in plants, including resistance to drought and salinity stresses, and are considered reliable targets for developing stress-tolerant crop plants in the future [27]. Consequently, it was no surprise that the number one cellular component affected by the two abiotic stresses in question, either under drought or salinity stress, was an integral component of a membrane in the transcriptome profile and cytoplasm in the proteome profile.

The oxidoreductases, transferases, and hydrolases classes of enzymes had the highest number of positive hits, similar to what we saw in Bittencourt et al. [17]. Within those classes, one can find several subclasses equally affected by drought and salinity stress. Several successful cases of resistance to abiotic stresses achieved by the overexpression of genes coding for oxidoreductases, transferases, or hydrolases enzymes are available in the scientific literature [28,29,30], showing that here also resides reliable targets to generate stress-tolerant crop plants.

The integration of transcriptomics, proteomics, and metabolomics datasets to gain further insights into the mechanism behind the response of young oil palm plants to drought stress used a much larger number of differentially expressed molecules compared to the MOI study carried out previously by Bittencourt et al. [17], when a total of 510 enzymes and 19 metabolites, differentially expressed under salinity stress, were used. Here, 1955 DE enzymes from transcriptomics analysis, 131 DE enzymes from proteomics analysis, and 269 differentially expressed metabolites (DEMs) underwent MOI analysis. The consequent result was a significant increase in the number of unique DE molecules in the most affected pathways.

The present MOI study showed that the Cysteine and methionine metabolism was the most affected pathway in the apical leaf of young oil palm plants under drought stress, with 47 unique DE molecules. In Bittencourt et al. [17], this pathway was the number one in the rank of affected ones, tied together with Glycolysis/Gluconeogenesis pathway (map00010), both with 20 unique DE molecules. The latter turned out to be the seventh most affected pathway in the list of the most affected ones in young oil palm plants under drought stress, with 30 unique DE molecules. Glycine, serine, and threonine metabolism (map00260) and Amino sugar and nucleotide sugar metabolism (map00520) were present in both lists of most affected pathways, with 34 and 35 unique DE molecules, respectively, in plants under drought stress, and 14 and 12 DE molecules, respectively, under salinity stress [17]. At last, in the present study, when performing the MOI analysis only with the molecules (proteins and metabolites) differentially expressed in both scenarios—drought and salinity stresses—the Cysteine and methionine metabolism pathway again turned out to be the most affected.

Even though this study identified other pathways, further discussion will concentrate only on cysteine and methionine metabolism. Cysteine and methionine are sulfur-containing amino acids. Besides being the metabolic sulfide donor for all cellular components containing reduced sulfur, cysteine is also a precursor of methionine, glutathione, phytochelatins, iron-sulfur clusters, vitamin cofactors, and multiple secondary metabolites, and the incorporation of sulfide into cysteine takes place in chloroplasts, mitochondria, and the cytoplasm [31]. Our results show that L-serine O-acetyltransferase, the first enzyme in cysteine biosynthesis, which catalyzes the synthesis of O-acetylserine (OAS) from acetyl-CoA (AcCoA) and L-serine (L-Ser), is upregulated in the apical leaf of young oil palm plants under saline (Log_2_(FC) = 3.4) as well as drought stress (Log_2_(FC) = 4.7). The same is true for the cysteine synthase, which catalyzes the synthesis of L-cysteine and acetate from OAS and hydrogen sulfide and showed a ~2-fold and ~6-fold increase, respectively, under saline and drought stresses.

Several enzymes from the omnipresent methionine salvage pathway (MSP), which uses dioxygen to regenerate methionine, associated with a ratchet-like step that prevents methionine back degradation [32], were differentially expressed in the apical leaf of young oil palm plants under saline and drought stresses. Enzymes from this pathway, such as S-adenosylmethionine synthetase, S-adenosylmethionine decarboxylase, and tyrosine aminotransferase, were positively regulated under both abiotic stresses. Meanwhile, 5-methylthioribose kinase and L-glutamine-4-(methylsulfanyl)-2-oxobutanoate aminotransferase were both downregulated. Resistance to salt and oxidative stress was achieved in Arabidopsis plants by the overexpression of a S-adenosylmethionine synthetase gene from salt-resistance sugar beet [33]. Barley stripe mosaic virus-based virus-induced gene silencing (BSMV-VIGS) of a S-adenosylmethionine synthetase gene in the Tibetan wild barley genotypes XZ5 severely compromised its resistance to both drought and salinity stress [34].

Besides mapping the commonalities in the molecular response of young oil palm plants to drought and salinity stresses, the present study also reported the molecules that displayed the opposite behavior when submitted separately to those two stresses. Stachyose synthetase (XP_010931766.1) is the enzyme that showed to most opposite behavior, downregulated under salt stress (Log_2_(FC) = −5.5) and upregulated in drought (Log_2_(FC) = 4.9). This enzyme catalyzes the reaction that produces myo-inositol plus stachyose from alpha-D-Galactosyl-(1->3)-1D-myo-inositol and Raffinose and was regulated positively in the drought-tolerant conifer *Larix olgensis* A. Henry under PEG-simulated drought stress [35]. According to Okemo et al. [36], the shoots of *Tripogon loliiformis*—an Australian resurrection plant—accumulated stachyose synthase transcripts and stachyose during drought stress. Stachyose triggers the formation of the hallmarks of plant apoptotic-like cell death in the desiccation-sensitive *Nicotiana benthamiana* but not the resilient *T. loliiformis*. Because of that, the stachyose synthase gene from this species is a potential candidate for improving stress resistance.

The present study, and the one by Bittencourt et al. [17], used transcriptome datasets from the apical leaf of young oil palm plants already showing extensive necrosis [20,23]. The reason stachyose synthase gene in *E. guineensis* increased approximately 32-fold under drought but decreased by almost 100% under saline stress, and considering that the transcriptome profiles used came from leaves with extensive necrosis, this is a question that needs further study to be answered.

The enzyme that showed to second most opposite behavior, downregulated under salt stress (Log_2_(FC) = −2.3) and upregulated in drought (Log_2_(FC) = 3.1), is a Topoisomerase II (XP_010931766.1). John et al. [37] over-expressed a topoisomerase II gene from tobacco in *Nicotiana tabaccum* and observed an increase in the resistance to salt stress.

The enzyme (XP_010934998.1) that showed the most opposite behavior, upregulated under salt stress (Log_2_(FC) = 1.9) and downregulated in drought (Log_2_(FC) = −3.1), is a member of the Cytochrome P450, E-class, group IV (IPR002403), protein family. Cytochrome P450s (CYPs) act as versatile catalysts and play a crucial role in the biosynthesis of secondary metabolites, antioxidants, and phytohormones in higher plants. The expression of several CYP genes is regulated in response to environmental stresses, with a role important in the crosstalk between abiotic and biotic stress responses, which make them candidate genes for engineering crop species resilient to biotic and abiotic stresses [38].

## 4. Materials and Methods

### 4.1. Plant Material, Growth Conditions, Experimental Design, and Drought Stress

The oil palm plants used in this study are clones of the ones used in the Bittencourt et al. [17] study. All plants—from both studies—came from the same embryogenic calluses. The young oil palm plants used in both studies were clones regenerated out of embryogenic calluses obtained from the leaves of an adult plant—genotype AM33, a Deli x Ghana from ASD Costa Rica; and were subjected to treatments when they were in the growth stage known as “bifid” saplings. Before starting the experiments, plants were standardized according to their developmental stage, size, and the number of leaves.

The experiment consisted of two water availability levels (field capacity—control and water deprivation—stressed), with four replicates in a completely randomized design. For the omics (transcriptomics, metabolomics, and proteomics) analysis described in the present study, we collected the apical leaves from control and stressed plants 14 days after imposing the treatments (DAT). See Rodrigues-Neto et al. [21] and Salgado et al. [23] for additional details regarding growth conditions, experimental design, drought stress, and the morphophysiological response of the oil palm plants.

### 4.2. Single-Omics Data Analysis, Functional Annotation, and Integratomics Analysis

Samples harvested from control and stressed plants were immediately immersed in liquid nitrogen and stored at −80 °C until RNA/Protein/Metabolite extraction; three replicates (plants) per treatment for RNA and protein extraction and four for the extraction of metabolites.

See Salgado et al. [22] for additional details on total RNA extraction, quality analysis, library preparation, and sequencing. The RNA-Seq raw sequence data are in the Sequence Read Archive (SRA) database of the National Center for Biotechnology Information (NCBI) under the BioProject number PRJNA573093. The transcriptomics analysis was performed using the OmicsBox platform—version 2.0.36 [39], as previously described [22]. The oil palm genome [18,19]—downloaded from NCBI (BioProject PRJNA268357; BioSample SAMN02981535) in September 2021—was the reference genome for RNA-Seq data alignment. The pairwise differential expression analysis between experimental conditions (Stressed Plants X Control) was performed as described [17].

The protein extraction protocol used here was the same used before [17]. The same happened for protein preparation and LC-MS/MS analysis at the GenOne company (Rio de Janeiro, RJ, Brazil). Data were acquired in biological triplicates using the the Xcalibur software (version 2.0.7) (Thermo Scientific, Waltham, MA, USA). Raw files were processed using the PatternLab for Proteomics V software [40], also as described by [17].

The metabolite extraction protocol used here was the same used by [17]. The DataAnalysis 4.2 software (Bruker Daltonics, Bremen, Germany) was the first used to analyze the raw data from UHPLC-MS as mzMXL files, and the pre-processing of data, peak detection, alignment of retention times and the statistical analysis at the pre-processing stage was similarly used by [17]. Then, a dataset was created from control and stressed plants. The pre-processed data (csv file) underwent analysis in the Statistical Analysis, MS Peaks to Pathway, and pathway analysis modules of the MetaboAnalyst 5.0 [41,42], exactly as previously described [17]. The differentially expressed peaks (DEPs) selected were those passing the criteria of false rate discovery (FDR) ≤ 0.05 and Log_2_ (fold change [FC]) ≠ 1.

The differentially expressed (DE) full-length ORFs and proteins identified in the OmicsBox and PatternLab V analysis underwent KOALA annotation before MOI analysis (Figure 1). The approach used to integrate the three omics was pathway-based mapping, performed using the Omics Fusion platform [43], as shown in Figure 1 and previously described [17].

### 4.3. Correlation Analysis of Differentially Expressed Molecules under Two Distinct Scenarios—Salinity and Drought Stresses

To perform a correlation analysis of differentially expressed (DE) full-length RNAs, proteins, and metabolites under two distinct scenarios, we used sets of data with the DE molecules from this present study and re-used the respective ones from [17]. First, to check the data distribution, we used the Data Overview module of Omics Fusion [43], the web platform for integrative analysis of Omics data, and then the Scatter Plot one for the correlation analysis between the sets of data—a pairwise combination of the different molecules and scenarios evaluated. The input data was the Log_2_(FC) from the DE molecules obtained from the single-omics analysis.

## 5. Conclusions

The SOA and MOI studies here reported generated new insights on the early response of young oil palm plants to drought stress, pointing out genes, proteins, metabolites, and pathways directly affected by this stress.

The MOI study performed using the molecules differentially expressed under drought (from this study) and salinity stress (from [14]) gave a unique opportunity to map the similarities and dissimilarities in the molecular response of those plants to those abiotic stresses of very high importance.

All MOI studies showed that cysteine and methionine metabolism was the most affected pathway, independent of the abiotic stress applied. This was also the case when only those molecules differently expressed under both abiotic stresses were considered.

The correlation analysis revealed that only 8.85% of those enzymes which were differently expressed under both stresses had different qualitative profiles, corroborating the already known fact that plant responses to drought and salinity show several similarities, with emphasis on the osmotic effect.

Finally, besides allowing us to map the pathways most affected by two abiotic stresses in the leaves of young oil palm plants, this study also generated a list of genes, proteins, and metabolites responsive to those stresses. This list can be used as valuable information to help those interested in the prospection and validation of candidate genes to promote resistance to both abiotic stresses once overexpressed in oil palm or other plant species.

## Figures and Tables

**Table 1:** Absolute numbers of all peptides and proteins identified via proteomics analysis in the leaves of young oil palm plants submitted to drought stress.

	Control	Stressed	Total
Peptide Spectrum Match (PSM)	6733	6495	13,065
Total number of peptides	3659	3824	5182
Number of unique peptides	2098	2088	3068
Total number of proteins entries	1353	1617	1859
Total number of proteins using the maximum parsimony criterion	762	934	1085

**Table 2:** List of the pathways most affected by drought stress—number of unique molecules—obtained via Multi-Omics Integration (MOI). Transcriptomics, proteomics, and metabolomics data from leaves of young oil palm plants 14 days after imposition of the treatments (control and water deprivation).

Pathway Name	Pathway ID	Occurrence of			Unique Molecules
		Transcripts	Proteins	Metabolites	
Cysteine and methionine metabolism	270	30	8	14	47
Purine metabolism	230	25	3	17	42
Porphyrin and chlorophyll metabolism	860	24	4	12	37
Amino sugar and nucleotide sugar metabolism	520	25	4	9	35
Glycine, serine, and threonine metabolism	260	24	7	9	34
Pyrimidine metabolism	240	17	1	16	33
Glycolysis/Gluconeogenesis	10	26	9	4	30
Phenylpropanoid biosynthesis	940	12	3	18	30
Pentose phosphate pathway	30	17	8	11	29
Arginine and proline metabolism	330	16	1	13	29
Aminoacyl-tRNA biosynthesis	970	13	1	15	29
Glyoxylate and dicarboxylate metabolism	630	17	8	8	28
Pyruvate metabolism	620	20	6	5	26
Starch and sucrose metabolism	500	22	5	3	26
Tyrosine metabolism	350	13	2	12	25
Phenylalanine, tyrosine, and tryptophan biosynthesis	400	17	1	8	25
Methane metabolism	680	16	5	8	24
Alanine, aspartate, and glutamate metabolism	250	18	5	6	24
Lysine degradation	310	14	3	9	24
Tryptophan metabolism	380	15	4	7	23
Terpenoid backbone biosynthesis	900	17	4	5	23
Carbon fixation in photosynthetic organisms	710	19	6	3	22
Citrate cycle (TCA cycle)	20	16	8	5	21
Arginine biosynthesis	220	9	3	11	21
Galactose metabolism	52	12	3	8	21
Valine, leucine, and isoleucine degradation	280	15	2	5	21
Carbon fixation pathways in prokaryotes	720	13	4	6	20
Glutathione metabolism	480	13	3	7	20
Glycerolipid metabolism	561	15	2	4	20
Ascorbate and aldarate metabolism	53	13	1	7	20

**Table 3:** Enzymes differentially expressed in the leaf of young oil palm plants under both drought and salinity stresses.

Protein ID	Gene ID *	KO Number	EC	Source		Profile		Fold Change		Description_KEGG
				Salt	Drought	Salt	Drought	Salt	Drought	
A0A6J0PH47	LOC105044080	K02641	1.18.1.2	Common	Common	Down	Down	−1.885	−3.730	ferredoxin-NADP+ reductase
B3TLY5	CAT2	K03781	1.11.1.6	Common	Common	Up	Up	1.834	3.177	catalase
B3TM49	LOC105035746	K08967	1.13.11.53	Only Control	Common	Down	Up	NA	3.277	1,2-dihydroxy-3-keto-5-methylthiopentene dioxygenase
			1.13.11.54							
A0A6I9QHS4			1.13.11.53			Down	Up	NA	2.463	
			1.13.11.54							
A0A6J0PS13	LOC105039298	K15918	2.7.1.31	Only Control	Only Control	Down	Down	NA	NA	D-glycerate 3-kinase
A0A6I9RVC5	LOC105053135	K05396	4.4.1.15	Only Control	Only Control	Down	Down	NA	NA	D-cysteine desulfhydrase
A0A6J0PP21	LOC105053549	K00434	1.11.1.11	Only Control	Only Stressed	Down	Up	NA	NA	L-ascorbate peroxidase
A0A6I9S451										
A0A6I9RWM2	LOC105053973	K00344	1.6.5.5	Only Control	Only Stressed	Down	Up	NA	NA	NADPH: quinone reductase
A0A6I9QWL2	LOC105040656	K03405	6.6.1.1	Only Stressed	Only Stressed	Up	Up	NA	NA	magnesium chelatase subunit I
A0A6I9QXL0	LOC105041662	K10525	5.3.99.6	Only Stressed	Only Stressed	Up	Up	NA	NA	allene oxide cyclase
A0A6J0PKN5	LOC105048171	K03405	6.6.1.1	Only Stressed	Only Stressed	Up	Up	NA	NA	magnesium chelatase subunit I
A0A6I9QUV8	LOC105040597	K00430	1.11.1.7	Only Stressed	Only Stressed	Up	Up	NA	NA	peroxidase
M1H922	LOC105057156	K13379	2.4.1.-	Only Stressed	Only Stressed	Up	Up	NA	NA	reversibly glycosylated polypeptide/UDP-arabinopyranose mutase
			5.4.99.30							
A0A6I9R7V0	LOC105045344	K25108	3.2.1.73	Only Stressed	Only Stressed	Up	Up	NA	NA	licheninase
A0A6I9R784	LOC105045348									
A0A6I9QEA6	LOC105033962									
A0A6I9R7N0	LOC105045343									
A0A6I9QD17	LOC105033964									

* Gene ID—In *Elaeis guineensis* genome at NCBI; KO—KEGG Orthology; EC—Enzyme Commission Number; Common—Present in both control and stressed; NA—Not Applicable; Up—Upregulated; Down—Downregulated.

**Table 4:** Molecules (full-length transcripts, metabolites, and proteins) from the Cysteine and methionine metabolism (map00270) pathway differentially expressed in the apical leaf of young oil palm plants under both drought and salinity stresses.

Name	Type	EC	KO	Salinity			Drought			Description
				logFC	Profile	Source	logFC	Profile	Source	
XP_010905665.1	TRNS	2.3.1.30	K00640	3.4	Up	NA	4.7	Up	NA	cysE; serine O-acetyltransferase
XP_010914049.1	TRNS	4.1.1.50	K01611	0.7	Up	NA	2.1	Up	NA	speD, AMD1; S-adenosylmethionine decarboxylase
XP_010915297.1	TRNS	2.5.1.47	K01738	0.9	Up	NA	2.5	Up	NA	cysK; cysteine synthase
XP_010920378.1	TRNS	2.6.1.1	K14454	1.0	Up	NA	3.8	Up	NA	GOT1; aspartate aminotransferase, cytoplasmic
XP_010921340.1	TRNS	2.5.1.6	K00789	1.4	Up	NA	2.4	Up	NA	metK, MAT; S-adenosylmethionine synthetase
XP_010924485.1	TRNS	2.3.1.30	K00640	−0.9	Down	NA	−0.7	Down	NA	cysE; serine O-acetyltransferase
XP_010927510.1	TRNS	1.1.1.95	K00058	−0.8	Down	NA	−1.2	Down	NA	serA, PHGDH; D-3-phosphoglycerate dehydrogenase/2-oxoglutarate reductase
XP_010928399.1	TRNS	2.7.1.100	K00899	−1.0	Down	NA	−2.5	Down	NA	mtnK; 5-methylthioribose kinase
XP_010931932.1	TRNS	6.3.2.3	K21456	−0.5	Down	NA	−1.1	Down	NA	GSS; glutathione synthase
XP_010938608.1	TRNS	2.6.1.44	K00830	−0.6	Down	NA	−3.2	Down	NA	AGXT; alanine-glyoxylate transaminase/serine-glyoxylate transaminase/serine-pyruvate transaminase
XP_010939394.1	TRNS	2.6.1.117	K23977	−0.5	Down	NA	−0.7	Down	NA	GTK; L-glutamine---4-(methylsulfanyl)-2-oxobutanoate aminotransferase
XP_010941130.1	TRNS	1.1.1.27	K00016	−3.4	Down	NA	−1.7	Down	NA	LDH, ldh; L-lactate dehydrogenase
XP_010942212.1	TRNS	2.5.1.16	K00797	−0.7	Down	NA	0.5	Up	NA	speE, SRM, SPE3; spermidine synthase
XP_010943220.1	TRNS	1.1.1.37	K00026	−0.6	Down	NA	−1.9	Down	NA	MDH2; malate dehydrogenase
XP_019711215.1	TRNS	2.6.1.5	K00815	0.8	Up	NA	1.0	Up	NA	TAT; tyrosine aminotransferase
C00051	METB	-	NA	0.3	Up	NA	0.9	Up	NA	Glutathione
C00049	METB	-	NA	1.1	Up	NA	4.6	Up	NA	L-Aspartate
A0A6I9QHS4	PROT	1.13.11.53	K08967	NA	NA	OC	1.3	Up	COM	1,2-dihydroxy-3-keto-5-methylthiopentene dioxygenase
		1.13.11.54								
A0A6I9RVC5	PROT	4.4.1.15	K05396	NA	NA	OC	NA	NA	OC	D-cysteine desulfhydrase
B3TM49	PROT	1.13.11.53	K08967	NA	NA	OC	1.7	Up	COM	1,2-dihydroxy-3-keto-5-methylthiopentene dioxygenase
		1.13.11.54								

NA—Not Applicable; OC—Only Control; COM—Common, present in the control as well as the stressed treatments.

## Data Availability

The datasets used and/or analyzed in the current study are available from the corresponding author on reasonable request.

## Supplementary Materials

The following supporting information can be downloaded at: https://www.mdpi.com/article/10.3390/plants11202786/s1, Table S1: Proteins from the oil palm genome differentially expressed (FDR ≤ 0.05) in the leaves of young oil palm plants under drought stress. FC—Fold Change, CPM—Count Per Million, FDR—False Discovery Rate, Kegg Orthology number, and E.C. number; Table S2: List of all peptides confidently identified in oil palm conditions; Table S3: List of all proteins confidently identified in oil palm conditions; Table S4: All proteins confidently identified in oil palm conditions with maximum parsimony criterion; Table S5: List of the proteins detected only in control biological condition; Table S6: List of the proteins detected only in stressed biological condition; Table S7: List of all differentially expressed proteins detected in both biological conditions (Stressed and Control) with statistical significance (FDR ≤ 0.05); Table S8: List of metabolites identified in the leaves of young oil palm plants submitted to drought stress via metabolomics analysis, after submitting the differentially expressed (DE) peaks to the pathway topology analysis module in MetaboAnalyst 5.0; Table S9: group of 554 enzymes differentially expressed under both abiotic stresses—drought and salinity.
